# Epidemiology and Genetics of Mucopolysaccharidosis Type VI in Russia

**DOI:** 10.3389/fmolb.2021.780184

**Published:** 2022-01-18

**Authors:** Elena Voskoboeva, Alla Semyachkina, Ochir Miklyaev, Amina Gamzatova, Svetlana Mikhaylova, Nato Vashakmadze, Galina Baydakova, Olga Omzar, Natalia Pichkur, Ekaterina Zakharova, Sergey Kutsev

**Affiliations:** ^1^ Federal State Budgetary Scientific Institution “Research Center for Medical Genetics”, Moscow, Russia; ^2^ Republican Center for Medical Genetics, Makhachkala, Russia; ^3^ Research and Clinical Institute of Pediatrics Named After Yuri Veltischev of the Pirogov Russian National Research Medical University of the Ministry of Health of the Russian Federation, Moscow, Russia; ^4^ Ministry of Health of the Russian Federation Research and Clinical Institute for Pediatrics of the Pirogov Russian National Research Medical University Detached Structural Unit Russian Children’s Clinical Hospital, Moscow, Russia; ^5^ Central Clinical Hospital of the Russian Academy of Sciences, Moscow, Russia; ^6^ Pediatrics Department of Pirogov Russian National Research Medical University, Moscow, Russia; ^7^ State Budgetary Healthcare institution of the Republic of Tyva “Perinatal Center of the Republic of Tyva”, Kyzyl, Russia; ^8^ National Children Hospital “OKHMATDET” of Ministry of Health of Ukraine, Kiev, Ukraine

**Keywords:** mucopolysaccharidosis type VI (MPS VI), Maroteaux-Lamy syndrome, arylsulfatase B, ARSB mutation, lysosomal enzyme, Russian Federation

## Abstract

Mucopolysaccharidosis VI (MPS VI) is an autosomal recessive lysosomal storage disease caused by mutations in the arylsulfatase B gene (*ARSB*) and consequent deficient activity of ARSB, a lysosomal enzyme involved in the glycosaminoglycan (s) (GAGs) metabolism. Here, we present the results of the study of *ARSB* DNA analysis in MPS VI patients in the Russian Federation (RF) and other republics of the Former Soviet Union. In a cohort of 68 patients (57 families) with MPS VI, a total of 28 different pathogenic alleles were found. The most prevalent nucleotide changes included NM_000046.5:c.194C>T and NM_000046.5:c.454C>T. Five pathogenic alleles were novel, not previously reported (NM_000046.5:c.304C>G, NM_000046.5:c.533A>G, NM_000046.5:c.941T>C, NM_000046.5:c.447_456del10, and NM_000046.5:c.990_10003del14). The nucleotide variant NM_000045.6:c.454C>T was the prevalent allele among Slavic Russian patients. The nucleotide variant NM_000045.6:c.194C>T was found only in MPS VI families from the Republic of Dagestan. Based on the analysis of dry blood spots (DBSs) collected from newborns in this RF region, we showed the frequency of this mutant allele in the Republic of Dagestan to be 0.01 corresponding to the MPS VI frequency of nearly 1:10,000, which is one of the highest worldwide. This may eventually make the selective asymptomatic carrier test and newborn screening highly feasible in this region of the country.

## Introduction

Mucopolysaccharidosis VI (MPS VI, OMIM: 253200) is a rare autosomal recessive lysosomal storage disorder (LSD) caused by mutations in the arylsulfatase B gene (*ARSB*) located on chromosome 5 (5q13–5q14) (Karageorgos et al., 2007). Pathogenic nucleotide variants in this gene result in reduced activity of arylsulfatase B (ASB, N-acetyl-galactosamine-4-sulfatase, EC 3.1.6.12), an enzyme required for the degradation of the glycosaminoglycans (GAGs) dermatan sulfate (DS) and chondroitin 4-sulfate (C4S). Dermatan sulfate is found in the connective tissue of various organs, the skin, tendons, blood vessels, and the respiratory tract, but mainly in heart valves. The accumulation of dermatan sulfate in lysosomes leads to irreversible cell damage and organ dysfunction. There are several forms of MPS VI, mild (attenuated), intermediate, and severe, based on the clinical features (Neufeld et al., 2001).

Birth prevalence has been reported to range from 1 in 43,261 live births in Turkish immigrants living in Germany to 1 in 1,505,160 live births in Sweden (Baehner et al., 2005; Malm et al., 2008)*.* Recently, detailed analysis of the epidemiology of the mucopolysaccharidoses in different countries has been performed. Along with MPS VII, MPS VI is a rare type MPS in most populations. However, in Brazil, Portugal ***,*** and British Columbia, the frequency of MPS VI is high in comparison to other MPSs. In Australia and Tunisia, the incidence of MPSVI is also higher than that in other countries (Khan et al., 2017).

To date, more than 200 pathogenic nucleotide variants in the *ARSB* gene have been identified (most of which are missense), suggesting a high molecular heterogeneity of MPS VI in many populations (http://www.uwcm.ac.uk).

Genotype–phenotype correlation for most MPS VI patients has been difficult to assess due to a large number of pathogenic nucleotide variants, which are often private or novel. Missense variants, which comprise the majority of mutations in MPS VI, have been found in patients with both severe and attenuated phenotypes (Karageorgos et al., 2007). Some mutations have been attributed to founders (Garrido et al., 2007; Jurecka et al., 2012; Costa-Motta et al., 2014; Jurecka et al., 2014; Zheng et al., 2014).

Specific treatment options available for this disorder are enzyme replacement therapy (ERT) and allogeneic hematopoietic stem cell transplantation (HSCT) (Giugliani et al., 2014; Taylor et al., 2019).

The aim of the study was to summarize the results of DNA analysis and investigate the variation in the pathogenic allele spectrum and the frequency in MPS VI patients in different regions and populations of the Russian Federation (RF) and other republics of the Former Soviet Union.

## Methods

### Patients

Patients were initially consulted at regional medical genetic consultations or the Scientific Clinical Institute of Pediatrics (Moscow) or the Russian Children’s Clinical Hospital (Moscow). Patients with suspected MPS VI were referred for confirmation of the diagnosis to the Laboratory of Hereditary Metabolic Diseases of Federal State Budgetary Scientific Institution “Medical Genetics Research Center” (Moscow, Russia).

A total of 75 patients were diagnosed with MPS VI during the period of 1989 through 2020. For 68 patients from 57 families, DNA samples were available and DNA analysis of the *ARSB* gene was performed. All patients were fully genotyped.

A total of 19 patients originated from the North Caucasus (Dagestan Republic, Ingushetia, Ossetia-Alania). Of them, 11 were Avars, one, Lak, two, Ingushes, four, Ossetians, and one, a descendant of a mixed marriage (Ossetian/Russian). One patient was Armenian, one, Azerbaijani, one, Belorussian, three, Ukrainians, one, Turkmenian, one, Kalmyk, three, Tuvans, and other 38, Russians. Russian patients were from different parts of the RF (central and eastern to Siberia and the Far East). The patients’ phenotypes varied from mild to severe. Informed consent was obtained from patients, their parents, or legal guardians.

### Biochemical Methods

ARSB activity was assayed in leukocytes or in dry blood spots (DBSs) by the fluorimetric or MS/MS methods (Blau et al., 2008; Kumar et al., 2015), and electrophoresis of urinary GAGs was performed according to the method described previously (Hopwood and Harrison, 1982).

### DNA Analysis

DNA extraction was carried out according to the manufacturer’s protocol using a DIAtomt DNA Prep100 kit (Isogene Lab. Ltd., Russia).

The eight exons and exon–intron boundaries of the *ARSB* gene were amplified from DNA samples. Primers and PCR reaction conditions have been previously described (Petry et al., 2003). Sequencing was performed according to the manufacturer’s protocol on an ABI Prism 3500XL (Applied Biosystems).

Blood specimens (unidentified) were provided by the neonatal screening center in Makhachkala (Republic of Dagestan). PCR-RFLP analysis using PspN4 I (GGN^∧^NCC) restriction endonuclease (SibEnzyme, Russia) was developed for the detection of NM_000046.5:c.194C>T mutation. To estimate the frequency of the NM_000046.5:c.194C>T mutation, 500 DNA samples were examined.

The frequency of the disorder was calculated from the Hardy–Weinberg equation: p^2^+ 2pq + q^2^ = 1. The confidence interval for frequencies was calculated by the Wilson method.

## Results

A cohort of 68 patients (57 families) from different regions of the Russian Federation and other former Soviet republics was investigated for mutations in the *ARSB* gene. A total of 28 special mutations in different combinations were revealed. Of them, 14 were missense mutations, five, nonsense mutations, four, small deletion, one, small insertion, and three, site-splicing mutations. Five mutant alleles were first described in this study: NM_000046.5:c.304C>G (NP_000037.2:p.Arg102Gly), NM_000046.5:c.533A>G (NP_000037.2:p.His178Arg), NM_000046.5:c.941T>c (NP_000037.2:p.Leu314Pro), c.447_456del10 (NP_000037.2:p.Gly149fs), and NM_000046.5:c.990_1003del14 (NP_000037.2:p.Phe331fs). The novel missense mutations (NP_000037.2:p.Arg102Gly, NP_000037.2:p.His178Arg, NP_000037.2:p.Leu314Pro) are likely to be pathogenic, predicted by the bioinformatics tools used, Mutation Taster, PolyPhen-2, SIFT, and PROVEAN. Mutations have also been classified according to the ACMG Criteria (Table 1, 2).

**TABLE 1:** Genotype and phenotype of patients with an indication of the place of residence and nationality.

Family	Patient	Nationality	Country or region of RF/federal districts	Phenotype	Genotype cDNA position (NM_000046.5) and amino acid alteration (NP_000037.2)	HGMD ID
1–9	G.S., G.Sh., S.M., H.H.,Sh.P., Sh.N., N.M., A.A., D.Sh, N.P., D.A.	Avar	The Republic of Dagestan/North Caucasian	mild	c.194C > T/c.194C > T p.Ser65Phe/p.Ser65Phe	CM990187
10	B.A.	Laks	The Republic of Dagestan/North Caucasian	mild	c.194C > T/c.194C > T p.Ser65Phe/p.Ser65Phe	CM990187
11	A.M.	Ingush	The Republic of Ingushetia/North Caucasian	severe	с.990_1003del14/c.990_1003del14 p.Phe331fs	novel
	A.H	Ingush	The Republic of Ingushetia/North Caucasian	severe	с.990_1003del14/c.990_1003del14 p.Phe331fs	novel
12	D.E.	Ossetian	Republic of North Ossetia-Alania/North Caucasian	severe	c.691-1G > A/c.691-1G > A	CS075068
13	М.A	Ossetian	Republic of North Ossetia-Alania/North Caucasian	severe	c.691-1G > A/c.691-1G > A	CS075068
14	T.A	Ossetian	Republic of North Ossetia-Alania/North Caucasian	severe	c.691-1G > A/c.691-1G > A	CS075068
	T.M	Ossetian	Republic of North Ossetia-Alania/North Caucasian	severe	c.691-1G > A/c.691-1G > A	CS075068
15	D.E.	Russian/Ossetian	Republic of North Ossetia-Alania/North Caucasian	intermediate	c.454C > T/c.691-1G > A p.Arg152Trp/c.691-1G > A	CM940116/CS075068
16	S.M	Tuvan	Tyva Republic/Siberian	severe	c.293T > C/c.293T > C p.Leu98Pro/p.Leu98Pro	CM003994
17	S.D	Tuvan	Tyva Republic/Siberian	severe	c.293T > C/c.293T > C p.Leu98Pro/Leu98Pro	CM003994
18	S.M	Tuvan	Tyva Republic/Siberian	severe	c.275C > A/c.293T > C p.Thr92Lys/p.Leu98Pro	CM074018/CM003994
19	Z.A	Kalmyk	Republic of Kalmykia/Southern	severe	c.943C > T/c.943C > T p.Arg315Ter/p.Arg315Ter	CM003996
20	L.Vic	Russian	Pskov region/Northwestern	mild	c.454C > T/c.941T > C p.Arg152Trp/p.Leu314Pro	CM940116/novel
	L.V.	Russian	Pskov region/Northwestern	mild	c.454C > T/c.941T > C p.Arg152Trp/p.Leu314Pro	CM940116/novel
21	T.A	Russian	Novgorod region/Northwestern	severe	c.1336+2T > G/c.1336+2T > G	CS040524
22	K.Z	Russian	St. Petersburg/Northwestern	severe	c.943C > T/c.943C > T p.Arg315Ter/p.Arg315Ter	CM003996
23	S.I	Russian	St. Petersburg/Northwestern	mild	c.629A > G/c.785dupA p.Tyr210Cys/p.Asn262fs	CM960081/CI146939
24	S.E	Russian	Murmansk region/Northwestern	n.d	c.237_243del/c.797A>C p.Val80fs/p.Tyr513Ter	CD941598/CM074042
25	Sh.S	Russian	Arkhangelsk region/Northwestern	severe	c.943C > T/c.1539C > G p.Arg315Ter/p.Tyr513Ter	CM003996/CM004000
26	I.T	Russian	Bryansk region/Central	mild	c.262C > T/c.454C > T p.Gln88Ter/p.Arg152Trp	CM1110928/CM940116
27	E.S.	Russian	Moscow region/Central	mild	c.454C > T/c.454C > T p.Arg152Trp/p.Arg152Trp	CM940116
28	L.D	Russian	Moscow region/Central	mild	c.454C > T/c.454C > T p.Arg152Trp/p.Arg152Trp	CM940116
29	M.E	Russian	Moscow region/Central	severe	c.1562G > A/c.1562G > A p.Cys521Tyr/p.Cys521Tyr	CM940121
30	L.S	Russian	Moscow region/Central	mild	c.454C > T/c.479G > A p.Arg152Trp/p.Arg152Trp	CM940116/CM940117
31	G.S	Russian	Moscow region/Central	mild	c.629A > G/с.941T > C p.Tyr210Cys/p.Leu314Pro	CM960081/novel
32	T.I	Russian	Moscow region/Central	mild	c.262C > T/c.275C > A p.Gln88Ter/p.Thr92Lys	CM1110928/CM074018
33	M.O	Russian	Moscow region/Central	intermediate	c.454C > T/c.1562G > A p.Arg152Trp/p.Cys521Tyr	CM960081/CM940121
34	K.Yu	Russian	Moscow region/Central	intermediate	c.454C > T/c.1562G > A p.Arg152Trp/p.Cys521Tyr	CM960081/CM940121
35	O.Z	Russian	Moscow/Central	mild	с.304C > G/c.797A > C p.Arg102Gly/p.Tyr266Ser	novel/CM074042
36	B.S.	Russian	Moscow/Central	intermediate	c.962T > C/c.962T > C p.Leu321Pro/p.Leu321Pro	CM940120
37	J.S	Russian	Nizhny Novgorod Region/Volga (Privolzhsky)	intermediate	c.629A > G/c.1562G > A p.Tyr210Cys/p.Cys521Tyr	CM960081/CM940121
38	M.A.	Russian	Nizhny Novgorod Region/Volga (Privolzhsky)	mild	c.454C > T/c.454C > T p.Arg152Trp/p.Arg152Trp	CM940116
	M.E.	Russian	Nizhny Novgorod Region/Volga (Privolzhsky)	mild	c.454C > T/c.454C > T p.Arg152Trp/p.Arg152Trp	CM940116
39	V.Al	Russian	Chuvash Republic/Volga (Privolzhsky)	mild	c.454C > T/c.533A > G p.Arg152Trp/p.His178Arg	CM940116/novel
	V.An	Russian	Chuvash Republic/Volga (Privolzhsky)	mild	c.454C > T/c.533A > G p.Arg152Trp/p.His178Arg	CM940116/novel
40	P.A	Russian	Chuvash Republic/Volga (Privolzhsky)	intermediate	c.454C > T/c.1562G > A p.Arg152Trp/p.Cys521Tyr	CM940116/CM940121
	P.V	Russian	Chuvash Republic/Volga (Privolzhsky)	intermediate	c.454C > T/c.1562G > A p.Arg152Trp/p.Cys521Tyr	CM940116/CM940121
41	S.D	Russian	Rostov region/Southern	mild	c.454C > T/c.1079T > C p.Arg152Trp/p.Leu360Pro	CM940116/CM003998
42	P.V.	Russian	Rostov region/Southern	mild	c.454C > T/c.1079T > C p.Arg152Trp/p.Leu360Pro	CM940116/CM003998
43	P.E	Russian	Chelyabinsk region/Ural	mild	c.262C > T/c.454C > T p.Gln88Ter/p.Arg152Trp	CM1110928/CM940116
	P.Yu	Russian	Chelyabinsk region/Ural	mild	c.262C > T/c.454C > T p.Gln88Ter/p.Arg152Trp	CM940116/CM1110928
44	F.A	Russian	Chelyabinsk region/Ural	intermediate	c.454C > T/c.943C > T p.Arg152Trp/p.Arg315Ter	CM940116/CM003996
45	M.Yu	Russian	Kurgan region/Ural	intermediate	c.454C > T/c.966G > A p.Arg152Trp/p/Trp322Ter	CM940116/CM074048
	M	Russian	Kurgan region/Ural	intermediate	c.454C > T/c.966G > A p.Arg152Trp/p.Trp322Ter	CM940116/CM074048
	M	Russian	Kurgan region/Ural	intermediate	c.454C > T/c.966G > A p.Arg152Trp/p.Trp322Ter	CM940116/CM074048
46	M.M	Russian	Tyumen region/Ural	mild	c.943C > T/c.943C > T p.Arg315Ter/p.Arg315Ter	CM003996
47	M.S	Russian	Novosibirsk region/Siberian	intermediate	c.454C > T/c.743del p.Arg152Trp/p.Pro248fs	CM940116/CD961786
48	V.M	Russian	Novosibirsk region/Siberian	intermediate	c.454C > T/c.943C > T p.Arg152Trp/p.Arg315Ter	CM940116/CM003996
49	K.D.	Russian	Irkutsk region/Siberian	intermediate	c.454C > T/c.966G > A p.Arg152Trp/p.Trp322Ter	CM940116/CM074048
50	M.V.	Russian	Khabarovsk region/Far Eastern	intermediate	c.454C > T/c.1562G > A p.Arg152Trp/p.Cys521Tyr	CM940116/CM940121
51	G.M.	Armenian	Ryazan Oblast/Central	severe	c.430G > A/c.430G > A p.Gly144Arg/p.Gly144Arg	CM940115
52	Sh.D	Azer	Moscow region/Central	severe	c.1213+5G > A/c.1213+5G > A	CS070362
53	M.D.	Turkmen	Turkmenia	severe	c.275C > A/c.275C > A p.Thr92Lys/p.Thr92Lys	CM074018
54	P.P	Ukrainian	Ukraine	intermediate	c.237_243del/c.454C>T p.Val80fs/p.Arg152Trp	CD941598/CM940116
55	H.M	Ukrainian	Ukraine	intermediate	c.245del/c.293T>C p.Leu82fs/p.Leu98Pro	CD004019/CM003994
56	K.D	Ukrainian	Ukraine	intermediate	c.454C>T/c.447_456del p.Arg152Trp/p.Gly149fs	CM940116/novel
57	M.T	Belorussian	Belorussian	intermediate	c.478C>T/c.479G>A p.Arg160Ter/p.Arg160Gln	CM940117/CM940118

**TABLE 2:** Annotation of the novel mutations of the *ARSB* gene.

Variant	Mutation type	dbSNP ID	Allele frequency (gnomAD)	Mutation Taster	PolyPhen-2	SIFT	PROVEAN	Criteria ACMG	Variant classification (ACMG category)
NM_000046.5:c.304C>G (NP_000037.2p:Arg102Gly)	missense	No	NA	Disease causing	deleterious	deleterious	Deleterious	PM1, PM2, PP2, PP3	Likely pathogenic
NM_000046.5:c.533A>G NP_000037.2p:His178Arg	missense	No	NA	Disease causing	deleterious	deleterious	Deleterious	PM1, PM2, PM5, PP2, PP3	Likely pathogenic
NM_000046.5:c.941T>C NP_000037.2p:Leu314Pro	missense	No	NA	Disease causing	deleterious	deleterious	Deleterious	PM1, PM2, PP2, PP3	Likely pathogenic
NM_000046.5:c.447_456del NP_000037.2:p.Gly149fs	Frame-shift deletion	No	NA	Disease causing	NA	NA	NA	PVS1, PM2, PP3	Pathogenic
NM_000046.5:c.990_1003del NP_000037.2:p.Phe331fs	Frame-shift deletion	No	NA	Disease causing	NA	NA	NA	PVS1, PM2, PP3	Pathogenic

**FIGURE 1:**
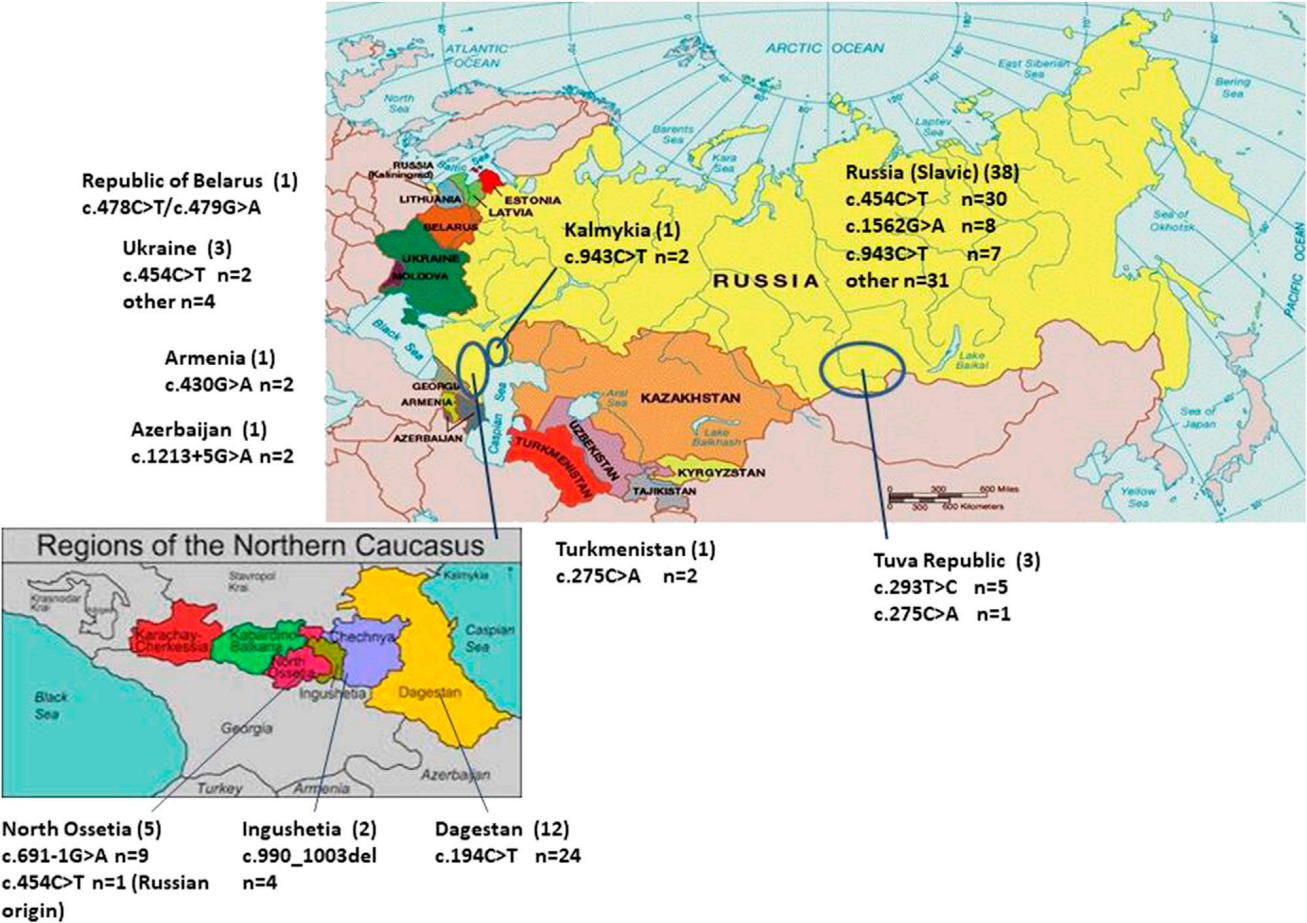
Distribution of the pathogenic *ARSB* alleles among patients from different population groups of Russia and other republics of the Former Soviet Union. The number of patients is indicated in brackets. n—number of pathogenic *ARSB* alleles.

The most prevalent mutations included NM_000046.5:c.194C> (NP_000037.2:p.Ser65Phe), NM_000046.5:c.454C>T (NP_000037.2:p.Arg152Trp), NM_000046.5:c.943C>T (NP_000037.2:p.Arg315Ter), and NM_000046.5:c.1562G>A (NP_000037.2:p.Cys521Tyr). The mutation NM_000046.5:c.454C>T previously described by Voskoboeva E. et al. (Voskoboeva et al., 1994) is common among Russian patients and was found in 39% of the alleles (30/76 alleles) in the present study. The second common mutation, NM_000046.5:c.194C>T, was found in the homozygous state only in patients from the Dagestan Republic. This mutation was described previously in an Italian family in a compound heterozygous state (Villani et al., 1999). The mutation NM_000046.5:c.943C>T was first detected in a Russian patient with a severe form of MPS VI (Voskoboeva et al., 2000). According to this study, NM_000046.5:c.943C>T accounts for 9.2% (7/76 alleles) among Russian patients. The mutation NM_000046.5:c.1562G>A was previously described in a French patient with a severe form of disease (Tomanin et al., 2018) and in 10.5% (8/76 alleles) of Russian patients (Figure 1).

### Clinical Phenotypes of the Patients

The age of onset ranged from 2 months to 5 years. The age at diagnosis ranged from 1 year to 24 years varied between 1 and 24 years. The following parameters were taken into account to rate disease severity: cardiac and respiratory involvement, ambulation or need for cervical surgery, number of surgeries, disease in joints, short stature, and the overall quality of life. Sixty percent of patients had received ERT. There was no detailed information about the clinical features of the patients as we mainly focused on the investigation of frequency and distribution mutations. Classification of subjects in severe, intermediate, or mild forms was based on the criteria of severity described below.

1. Severe form: the age at onset of symptoms less than 36 months, severe progression of symptoms, patients who evolved to death at less than 10 years of age, and patients with a height of less than 90 cm at the age of 10 years;
2. Intermediate form: age at onset of symptoms over 36 months, a height less than 140 cm at the last evaluation, and survival above 20 years of age.
3. Mild form: age at onset of symptoms over 36 months, the absence of mental retardation, a height above 140 cm, and survival to more than 20 years of age.

### Geographic Distribution

Clear ethnicity-related peculiarities were observed for some of the mutations. In the central south-eastern and eastern parts of the RF prevailed NM_000046.5:c.454C>T, NM_000046.5:c.943C>T, NM_000046.5:c.1562G>A among Russian patients of Slavic origin.

The nucleotide substitution NM_000046.5:c.194C>T was found only in 12 patients (10 families) from the Dagestan Republic (the south part of the RF). This variant has never been met in a healthy population. All the affected individuals originating from this region were found to be homozygous and were born from consanguineous parents (first cousins). The phenotype was attenuated in all patients with this mutation. In two Ingush families with severe phenotypes, a novel mutation NM_000046.5:c.990_1003del14 in a homozygous state was found, with parents also consanguineous.

In the Republic of North Ossetia-Alania, four patients from three family families had a severe form of MPS VI and were homozygous for site-splice mutation NM_000046.5:c.691-1G>A. One patient from a mixed marriage had an intermediate phenotype and was genotyped as NM_000046.5:c.[454C>T];[691-1G>A].

In the Tuva Republic, two severe patients were homozygous for NM_000046.5:c.293T>C and one more severe patient was compound for NM_000046.5:c.293T>C and NM_000046.5:c.275C>A.

In patients from Belorussia, Armenia, Azerbaijan, and Turkmenia, single unique nucleotide changes were detected. All patients, except Belorussian, were homozygotes for the pathogenic alleles found. In two from three Ukrainian patients, the allele NM_000046.5:c.454C>T was found in the heterozygote state; their second alleles were unique ones (Figure 1; Table 1).

## MPS VI Frequency in the Republic of Dagestan

For estimation of MPS VI frequency in the Republic of Dagestan, 500 DBS specimens collected from newborns in a neonatal screening center of Makhachkala (capital of the Republic of Dagestan) were analyzed. For the detection of the c.194C > T mutation, a PCR-RFLP analysis was developed using the restriction endonuclease PspN4 I (GGN ^ NCC). In the control, five fragments are formed: 74, 72, 18, 16, and 6 bp. In the presence of the mutation, the restriction site disappears, with the formation of only four fragments: 147, 18, 16, and 6 bp (data not shown).

A total of 10 mutation carriers were detected. Thus, the frequency of the mutant allele NM_000046.5:c.194C>T in the Republic of Dagestan was 0.01 (95% confidence interval: 0.00544÷0.018309; the estimated frequency of MPS VI (q^2^) was 0.0001 (95% confidence interval: 0.00001765÷0.00056627) or 1:10,000 (95% confidence interval: 1:2,500÷1:100,000). This frequency is one of the highest worldwide.

## Discussion

In a cohort of 68 patients from the RF, 28 different mutations were identified. Five mutations were first described in this study: NM_000046.5:c.304C>G, NM_000046.5:c.941T>C, NM_000046.5:c.533A>G, NM_447_456del10, and NM_000046.5:c.990_1003del14. Mutations NM_000046.5:c.304C>G, NM_000046.5:c.533A>G, NM_000046.5:c.447_456del10 were associated with intermediate phenotypes, NM_000046.5:c.990_1003del14 were found in two severely affected patients, and c.941T > C was associated with a mild form of disease.

Among patients of Slavic Russian origin, the pathogenic nucleotide variant NM_000046.5:c.454C>T was common and nucleotide changes NM_000046.5:c.1562G>A, NM_000046.5.c:943C>T were most frequent and amount for 39%, 10.5%, and 9.2%, respectively (Figure 1; Table 1).

**FIGURE 2:**
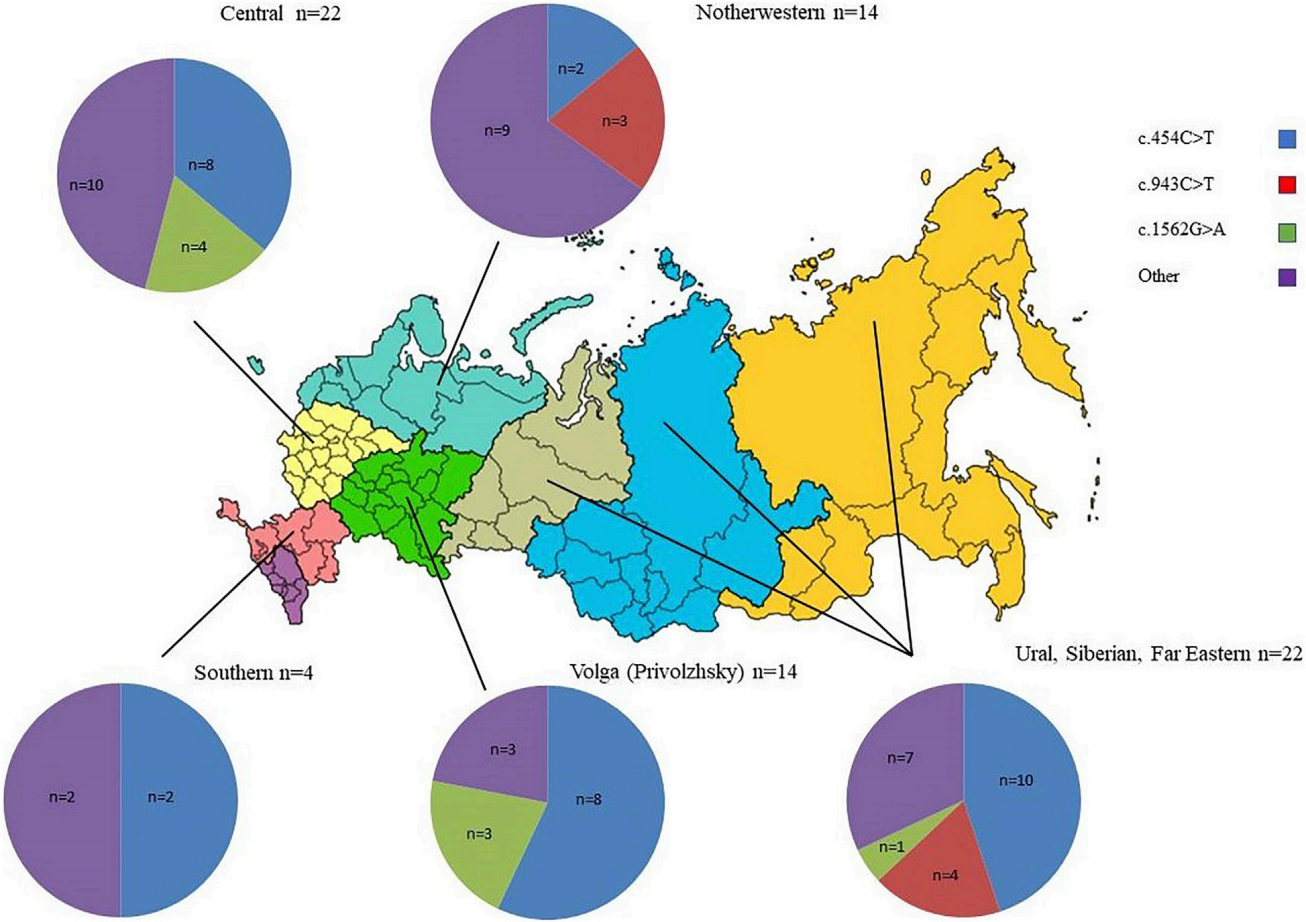
Distribution of frequent pathogenic *ARSB* alleles among Slavic Russian patients from different districts of the RF. n—number of *ARSB* alleles.

The high prevalence of the NM_000046.5:c.454C>T pathogenic allele (42–43%) observed in patients of Slavic origin (Russian, Belarus, Poland). The frequency of this allele in a healthy population (according to gnomAD) is 0.009289%. The observed high NM_000046.5:c.454C>T carrier frequency (0.6%) in the Lithuanian population indicates a possible founder effect in this region (Jurecka et al., 2012). Although a more recent study suggests that NM_000046.5:c.454C>T might be of Slavic and not Lithuanian origin (Jurecka et al., 2014). Patients homozygous for this mutation originated from the central part and Volga region of the RF, while this mutation in the compound-heterozygous state is described in Siberia and northern and southern parts of the RF (Figure 2; Table 1).

The pathogenic allele mutation NM_000046.5:c.943C>T was first described in a heterozygous state in our patient with the severe form of MPS VI (Voskoboeva et al., 2000), whose genotype was NM_000046.5:c.[943C>T];[1539C>G]. This Russian patient was the resident of Arkhangelsk oblast – the North northern part of the RF. Then, the mutation NM_000046.5:c.943C>T was found in five other patients. Three patients were homozygous for NM_000046.5c:943C>T and had a severe form of disease. Two of them were Russian and one, Kalmyk. Two other patients were heterozygous for NM_000046.5c:943C>T and had an intermediate form of MPS VI. Their second mutant allele was NM_000046.5c:454C>T. Interestingly, according to recent investigations, the mutation NM_000046.5c:943C>T is most common in Italy (Tomanin et al., 2018). We observed the absence of founder effects and an even distribution across the territory of the RF from the North part to the Ural-Siberian region (Table 1; Figure 2).

The pathogenic allele NM_000046.5c:1562G>A was thoroughly described in a patient with a severe form of the disease (Isbrandt et al., 1994). This nucleotide variant was found in six of our patients in a heterozygous form and in one patient in a homozygous form. For five heterozygous patients, the second allele was NM_000046.5c:454C>T. One patient was compound for NM_000046.5c:1562G>A and NM_000046.5c:629C>T. All patients had an intermediate phenotype. All patients were Russian, and six originated from the European part of RF. No data about founder effects for this mutation were received (Table 1; Figure 2).

The intronic variant NM_000046.5c:691-1G>A and the missense nucleotide change NM_000046.5c:293T>C accumulated in patients from Ossetia-Alania and the Tuva Republic, respectively (Figure 1).

The NM_000046.5c:691-1G>A nucleotide variant has previously been described in one patient as private. This allele is resulting results in a 27-base deletion and in-frame deletion of nine amino acids (Karageorgos et al., 2007). The North Ossetia is a republic within the Russian Federation in the North Caucasus. The main population of the republic is Ossetians – Iranian-speaking people, descendants of the Alans. Four Ossetian patients from three different families were homozygous for NM_000046.5c:691-1G>A and had severe phenotypes of MPS VI. One more patient was a descendant of Russian-Ossetian marriage. Her genotype was NM_000046.5c:[691-1G>A];[454C>T], and she has an intermediate form of the disease (Table 1). The intra-national marriages still prevail in the republic. Also, the predominance of the allele NM_000046.5c:691-1G>A probably indicates the founder effect of the NM_000046.5c:691-1G>A mutation in the Republic of Ossetia-Alania.

Despite the doubts concerning the damaging effect of the mutation NM_000046.5c:293T>C, we confidently presume it to be deleterious. First, the mutation NM_000046.5c:293T>C was described in a heterozygote at a heterozygous state in a patient from Ukraine (Voskoboeva et al., 2000). In three patients from Tuva, the mutation NM_000046.5c:293T>C was detected twice in a homozygous state and once in combination with mutation NM_000046.5c:275C>A. No other nucleotide changes in the *ARSB* gene were found in the patients. All patients were Tuvans. Tuvans are native people of Tuva Republic—subject of the Russian Federation in the Siberian Federal District (Figure 1). At present, Tuvans, unlike other peoples of the Altai-Sayan Upland, are characterized by endogamy—marriage within the limits of their ethnic group. Tuvans people have Turkic origin. It is unknown whether the Ukranian patient, in whom the mutation NM_000046.5c:293T>C was first discovered, had Turkic roots, but it cannot be excluded. Although there are little data so far, we assume a possible founder effect for the mutation NM_000046.5c:293T>C among Tuvans.

The mutant allele NM_000046.5c:194C>T was found only in patients from the Republic of Dagestan. Clinical phenotypes of these patients could be classified as intermediate or mild forms. All patients except one were Avars, and one patient was Lak (Table 1; Figure 1). The Republic of Dagestan is a federal subject of Russian Federation, located in the North Caucasus region. With a population of 2,910,249, the live birth per year is 47,690. The Republic of Dagestan is a highly ethnically diverse and the most heterogeneous region of Russia. Its ethnic majorities are the Avars, Dargins, Kumyks, Lezgians, Laks, Azerbaijanis, Tabasarans, and Chechens. Ethnic Russians comprise no more than about 3.6% of Dagestan’s total population (Bulayeva, 2006). Avars are one of the most numerous and ancient peoples, and their ancestors lived in the Caucasus in the Neolithic period. These people practically do not assimilate with other people: marriages of Avars with Russians or representatives of other Caucasian nationalities are very rare. Genetic diversity analysis showed that Dagestan ethnic populations are ancient, highly isolated populations with 85–97% the rate of the endogamy and inbreeding coefficient F = 0.010–0.015. Many Dagestan populations have very high prevalence of certain diseases such as mental retardation and progressive muscular dystrophy (Bulayeva, 2006).

A total of 12 patients from 10 families (18% of all patients) were originated from the Republic of Dagestan. All of them are homozygous for the NM_000046.5c:194C>T mutation. The frequency of the mutant allele NM_000046.5c:194C>T in the Republic of Dagestan was 0.01 (95% confidence interval: 0.00544÷0.018309), and the estimated frequency of MPS VI (q2) was 0.0001 (95% confidence interval: 0.00001765÷0.00056627) or 1:10,000 (95% confidence interval: 1:2,500÷1:100,000). This frequency is one of the highest in the world.

Obviously, given the very small number of patients examined and the low incidence of the disease, it is difficult to draw unambiguous conclusions. However, we allowed ourselves to speculate and identify trends in the distribution of unique pathogenic *ARSB* alleles in certain population groups of the RF.

To highlight some of the findings, our data show that 1. an accumulation of NM_000046.5c:454C>T mutation among Russian patients was detected, which is probably attributed to the founder effect. This is in agreement with the results of earlier studies (Jurecka et al., 2012; Jurecka et al., 2014); 2. NM_000046.5c:454C>T mutation has not been detected among patients from other ethnic groups. The prevalence of their unique alleles was detected among these patients. The prevalent mutations were NM_000046.5c:194C>T among patients from Dagestan, NM_000046.5c:293T>C among patients from Tuva, and NM_000046.5c:691-1G>A among patients from Ossetia; 3. allele frequency in the Republic of Dagestan was evaluated.

This study also has a number of limitations: 1. estimates of the frequencies of unique alleles in the populations examined may be overestimated because of few patients diagnosed. The assumption of the founder effect should be supported by additional studies. It is possible that the accumulation of certain alleles in the examined populations is related only to the frequency of consanguineous marriages. Either the mutations detected are “hot-spot”. 2. Analysis of novel mutations was performed only in silico. A functional analysis for detectable mutations in the *ARSB* gene, especially missense variants, is required to evaluate their actual effect on enzyme functions.

The results of our study demonstrate an uneven distribution of MPS VI in the Russian Federation. The high frequency of consanguineous marriages, which persist in some populations to this day, leads to the accumulation and spread of pathogenic alleles and an increase in the incidence of the disease within such populations. High risk and newborn screening for MPS VI based on frequency of disease in different regions could be discussed for early detection and start of treatment.

## Data Availability

The original contributions presented in the study are included in the article/Supplementary Material, and further inquiries can be directed to the corresponding author.

## Abbreviations

ARSB, arylsulfatase B; ERT, enzyme replacement therapy; GAG, glycosaminoglycan; HGMD, Human Gene Mutation database; LSD, lysosomal storage disorder; MPS, mucopolysaccharidosis.
